# Cancer stem cells from epithelial ovarian cancer patients privilege oxidative phosphorylation, and resist glucose deprivation

**DOI:** 10.18632/oncotarget.2010

**Published:** 2014-05-26

**Authors:** Anna Pastò, Chiara Bellio, Giorgia Pilotto, Vincenzo Ciminale, Micol Silic-Benussi, Giulia Guzzo, Andrea Rasola, Chiara Frasson, Giorgia Nardo, Elisabetta Zulato, Maria Ornella Nicoletto, Mariangela Manicone, Stefano Indraccolo, Alberto Amadori

**Affiliations:** ^1^ Department of Surgery, Oncology, and Gastroenterology, Oncology Section, University of Padova, Padova, Italy; ^2^ Istituto Oncologico Veneto-IRCCS (IOV), Padova, Italy; ^3^ Department of Biomedical Sciences, University of Padova, Padova, Italy; ^4^ Department of Woman and Child Health, Laboratory of Hemato-Oncology, University of Padova, Padova, Italy

**Keywords:** Ovarian cancer, Cancer Stem Cells, metabolism, glucose, Warburg effect

## Abstract

We investigated the metabolic profile of cancer stem cells (CSC) isolated from patients with epithelial ovarian cancer. CSC overexpressed genes associated with glucose uptake, oxidative phosphorylation (OXPHOS), and fatty acid β-oxidation, indicating higher ability to direct pyruvate towards the Krebs cycle. Consistent with a metabolic profile dominated by OXPHOS, the CSC showed higher mitochondrial reactive oxygen species (ROS) production and elevated membrane potential, and underwent apoptosis upon inhibition of the mitochondrial respiratory chain. The CSC also had a high rate of pentose phosphate pathway (PPP) activity, which is not typical of cells privileging OXPHOS over glycolysis, and may rather reflect the PPP role in recharging scavenging enzymes. Furthermore, CSC resisted *in vitro* and *in vivo* glucose deprivation, while maintaining their CSC phenotype and OXPHOS profile. These observations may explain the CSC resistance to anti-angiogenic therapies, and indicate this peculiar metabolic profile as a possible target of novel treatment strategies.

## INTRODUCTION

Cancer cells, unlike normal cells, privilege conversion of pyruvate to lactic acid for ATP generation rather than mitochondrial oxidative phosphorylation even in the presence of oxygen [1]: this peculiar metabolic profile is termed “aerobic glycolysis” or the “Warburg effect”. Although cancer stem cells (CSC) have been identified in virtually all malignancies [2-4], several key aspects of their physiology remain to be understood, as outlined by Clevers in his seminal review [5]. In particular, little if anything is known about the metabolic profile of CSC, and it is unclear whether CSC also present a prominent Warburg effect. Recent studies in a glioma cell line showed that cells exhibiting a CSC phenotype display some different metabolic properties, compared to their non-stem counterpart [6].

We investigated the metabolic profile of epithelial ovarian cancer (EOC) cells and their CSC population. EOC is a very malignant neoplasm, accounting for 5% of cancer mortality in women [7]. Ovarian cancer cells are easy to harvest *ex vivo* thanks to the expression of surface markers, which allow their isolation without *in vitro* manipulations that may alter their physiologic *status.* Furthermore, EOC effusion cells may be studied as single tumor cell suspensions in the absence of conditions that may alter their metabolism, such as hypoxia. It is well-known, in fact, that hypoxia has a strong influence on the growth properties of solid tumors, and the combination of hypoxia and nutrient deprivation in some tumor areas can affect functional parameters, such as metabolism and mitochondrial function [8, 9]. Here we show that an *ex vivo* isolated population of EOC cells co-expressing CD44 and CD117, the two critical markers of CSC, shows a metabolic profile characterized by high glucose uptake and preferential fuelling of glucose into oxidative phosphorylation (OXPHOS) and the pentose phosphate pathway. Notwithstanding, these cells resist *in vitro* and *in vivo* glucose deprivation while fully maintaining their OXPHOS and CSC properties.

## RESULTS

### CD44^+^CD117^+^ cells from ascitic effusions of EOC patients meet the hallmarks of canonical CSC

**Figure 1:**
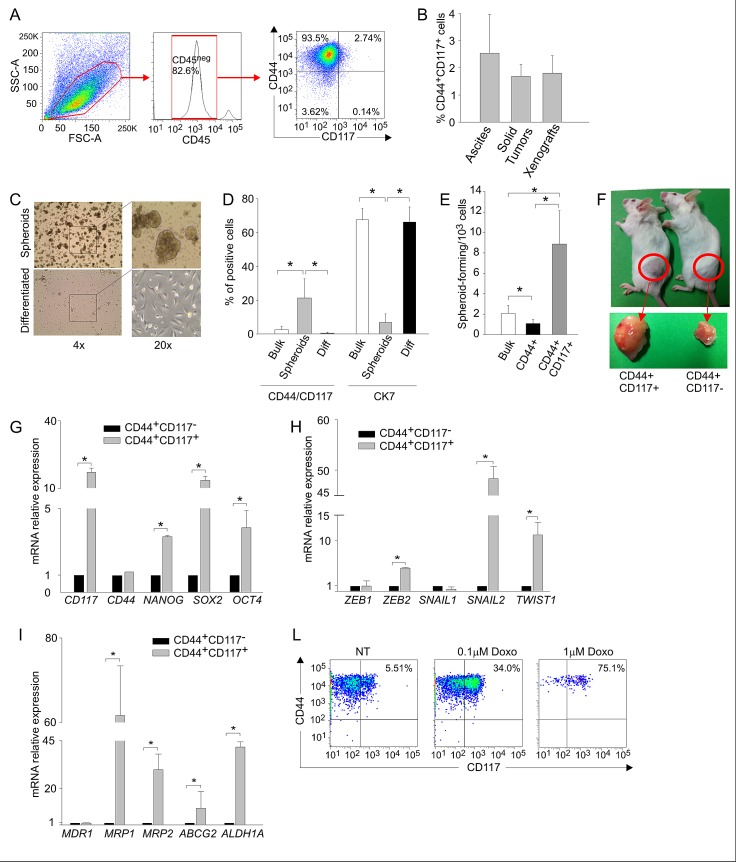
CD44^+^CD117^+^ cells from ovarian cancer effusions show a phenotypic, molecular and functional profile compatible with a canonical CSC population A. Cytofluorimetric analysis of a representative sample of ascitic effusion cells from an EOC–bearing patient. The expression of CD117 and CD44 was evaluated on CD45^neg^ cells, thus excluding contaminating CD45^+^ myeloid cells (middle panel). B. Percentage of CD44^+^CD117^+^ cells in EOC ascitic effusions (n=45), solid EOC tumors (n=6), and primary xenografts derived from injection of EOC effusion cells into immunodeficient mice (n=12). The graph shows mean percentages ± SD. C. Spheroid formation by EOC effusion cells cultured for 10 days in FBS-free RPMI enriched with EGF and bFGF (upper panels) followed by 10 days in complete RPMI to induce differentiation (lower panels). The results are representative of 5 experiments. D. FACS analysis of CD44/CD117 and CK7 expression in EOC effusion cells (Bulk), spheroids obtained after 10 days' culture in the absence of FBS (Spheroids), and after 10 days of culture in differentiating conditions (Diff). The graph shows mean percentages of positive cells ± SD measured in 10 experiments. *p < 0.05. E. Spheroid-forming cell frequency, calculated by extreme limiting dilution analysis (ELDA) and expressed as the number of spheroid-forming cells/10^3^ cells. ELDA was performed on unsorted cells (bulk), and on FACS-sorted CD44^+^CD117^+^ and CD44^+^CD117^−^ cells. Shown are mean spheroid-forming cell frequencies ± SD calculated from 3 consecutive experiments. *p < 0.05. F. Tumor generation in RAG-2γ^−/−^ mice injected s.c. with 1 × 10^5^ FACS-purified CD44^+^CD117^+^ cells (left) or CD44^+^CD117^−^ cells (right) from EOC ascitic effusions. G. qRT-PCR analysis of stemness-associated genes in FACS-sorted CD44^+^CD117^+^ and CD44^+^CD117^−^ cells from EOC ascitic effusions. The relative expression of each mRNA in CD44^+^CD117^+^ cells compared to CD44^+^CD117^−^ cells was calculated as described in the *Supplemental Data*. Shown are mean values ± SD measured in ten samples. *p < 0.05. H. qRT-PCR analysis of genes coding for transcription factors involved in Epitelial-to-Mesenchymal Transition in FACS-sorted CD44^+^CD117^+^ and CD44^+^CD117^−^ cells from EOC effusion samples. Shown are mean relative expression values in CD44^+^CD117^+^ cells compared to CD44^+^CD117^−^ cells ± SD measured in ten samples. *p < 0.05. I. qRT-PCR analysis of the expression of multidrug resistance pumps and detoxifying enzymes in FACS-sorted CD44^+^CD117^+^ and CD44^+^CD117^−^ cells from EOC effusion samples. Shown are mean relative expression values in CD44^+^CD117^+^ cells compared to CD44^+^CD117^−^ cells ± SD measured in ten samples. *p < 0.05. L. Flow cytometry analysis of CD44/CD117 expression in EOC effusion cells incubated with different concentrations of Doxorubicin for 48 hr.

Previous studies identified the co-expression of CD44 and CD117 as a marker of ovarian CSC [10, 11]. Before investigating the metabolic profile of this subset, we tested whether these markers identified CSC cells in ascitic effusions from EOC patients. As shown in Figures 1A and 1B, CD44^+^CD117^+^ cells accounted for a small percentage of the neoplastic population (2.5 ± 1.4%; range 0.2-5.0%). A similar percentage was found in EOC masses (Figure 1B), thus indicating that ascitic effusions mirror the composition of solid tumors. This percentage of CD44^+^CD117^+^ cells was also maintained after xenotransplantation of ascitic effusion cells into immunodeficient mice (Figure 1B).

CD44^+^CD117^+^ cells displayed canonical hallmarks of CSC cells. EOC effusion cells formed spheroids when cultured in the absence of serum (Figure 1C), a phenomenon peculiar to CSC from different cancer histotypes [3, 4]. Spheroid formation was associated with a significant increase in the percentage of CD44^+^CD117^+^ cells and a reduction in the expression of the differentiation marker cytokeratin-7 (CK7) (Figure 1D), whereas culture in the presence of serum resulted in a significant increase in CK7 expression, and a decrease in the percentage of CD44^+^CD117^+^ cells (Figure 1D). Moreover, spheroid-forming cells, as judged by extreme limiting dilution analysis (ELDA), were enriched in the double-positive population (Figure 1E). It is well-known that CSC present high tumor-initiating capacity. As shown in Table S1, 7 out of 12 RAG-2γ^−/−^ mice injected intra-peritoneally (i.p.) with 5 × 10^3^ purified CD44^+^CD117^+^ cells developed tumors, whereas no tumors were observed in 10 mice injected with the same amount of CD44^+^CD117^−^ cells; tumors could only be obtained when the number of inoculated CD44^+^CD117^−^ cells was increased by 100-fold (Table S1). When mice were injected subcutaneously (s.c.) with 1 × 10^5^ cells, the tumors generated by CD44^+^CD117^+^ cells were far larger than those generated by the injection of CD44^+^CD117^−^ cells (Figure 1F).

We also compared the expression of several stemness-related genes and genes associated with epithelial-to-mesenchymal transition (EMT). In agreement with previous data [12, 13], FACS-purified CD44^+^CD117^+^ cells displayed higher expression of all the stemness genes examined (Figure 1G), as well as of EMT-associated genes (Figure 1H). It is known that CSC are resistant to chemotherapy, in part due to the expression of drug-extruding pumps and detoxifying enzymes [14]. Compared to single-positive cells, CD44^+^CD117^+^ cells displayed higher expression of *MRP1*, *MRP2* and *ABCG2* pumps, as well as of *ALDH1A* (Figure 1I), a detoxifying enzyme which is also considered as a canonical marker of CSC [15]. This observation was supported by the finding that the percentage of CD44^+^CD117^+^ cells increased dramatically following *in vitro* incubation of EOC effusion cells with Doxorubicin (Figure 1L). Altogether, these results indicate that the CD44^+^CD117^+^ cells represent a *bona fide* CSC population in EOC ascitic effusions.

### Ovarian CSC show a peculiar expression profile of glucose metabolism- and fatty acid β-oxidation-associated enzymes

We next compared the *ex vivo* metabolic profiles of FACS-purified CD44^+^CD117^+^ and CD44^+^CD117^−^ cells by examining the expression of a panel of genes involved in key metabolic pathways, including glucose metabolism, the tricarboxylic acid (TCA) cycle, the electron transport chain (ETC), the pentose phosphate pathway (PPP), and fatty acid β-oxidation.

**Figure 2:**
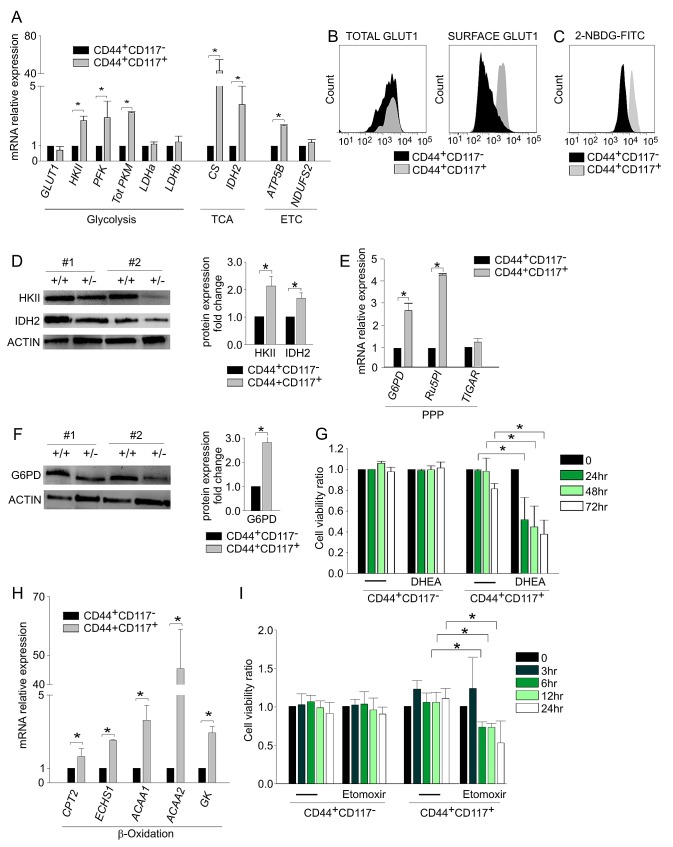
CD44^+^CD117^+^ ovarian cancer cells show a different profile of glucose metabolism- and fatty acid β-oxidation-associated enzymes A. FACS-sorted CD44^+^CD117^+^ and CD44^+^CD117^−^ cells from EOC ascitic fluid samples were analysed by qRT-PCR for the expression of key enzymes of glucose metabolism, the tri-carboxylic acid (TCA) cycle and the electron transport chain (ETC). Shown are mean relative expression values in CD44^+^CD117^+^ cells compared to CD44^+^CD117^−^ cells ± SD measured in ten samples. *p < 0.05. B. Total and surface expression of the glucose transporter GLUT1 was evaluated by flow cytometry in permeabilized (left panel) and non-permeabilized (right panel) EOC effusion cells. One representative experiment out of five is shown. C. Flow cytometry analysis of fluorescent glucose uptake by CD44^+^CD117^+^ and CD44^+^CD117^−^ cells from EOC ascitic effusions. The cells were labelled with anti-CD44 and anti-CD117 antibodies, and 2-NBDG-FITC was added; fluorescence intensity was recorded after 1 min. One representative experiment out of eight is shown. D. WB analysis of HKII and IDH2 expression in FACS-sorted CD44^+^CD117^+^ and CD44^+^CD117^−^ cells from EOC effusions. Results in two representative samples are shown on the left; +/+ denotes CD44^+^CD117^+^ cells, and +/− indicates CD44^+^CD117^−^ cells. Signal intensities of the HKII and IDH2 bands were quantitated by scanning densitometry and normalized against the actin signal. Expression ratios were calculated by dividing normalized signal intensity values obtained for CD44^+^CD117^+^ cells by those obtained for CD44^+^CD117^−^ cells. The graph shows mean expression ratios ± SD from three experiments. *p < 0.05. E. FACS-sorted CD44^+^CD117^+^ and CD44^+^CD117^−^ cells from EOC ascitic fluid samples were analysed by qRT-PCR for the expression of some key enzymes of the Pentose Phosphate Pathway (PPP). Shown are mean relative expression values in CD44^+^CD117^+^ cells compared to CD44^+^CD117^−^ cells ± SD measured in ten samples. *p < 0.05. F. WB analysis of G6PD expression in FACS-sorted CD44^+^CD117^+^ and CD44^+^CD117^−^ cells from EOC ascitic effusions. Results obtained for two representative samples are shown on the left; +/+ and +/− denote CSC and non-CSC subsets, respectively. The ratio of G6PD expression in CD44^+^CD117^+^ cells vs. CD44^+^CD117^−^ cells was calculated as described in the legend to Fig. 2D. The graph shows the mean expression ratio ± SD calculated from three experiments. *p < 0.05. G. Unfractionated EOC effusion cells were incubated with the PPP inhibitor DHEA, and the viability of CD44^+^CD117^+^ and CD44^+^CD117^−^ cells was analysed at different times by Annexin V/PI staining. The graph shows mean cell viability ratios ± SD measured in three consecutive experiments (calculated as detailed in the *Supplemental Data*). *p < 0.05. H. FACS-sorted CD44^+^CD117^+^ and CD44^+^CD117^−^ cells from EOC effusion samples were analysed by qRT-PCR for the expression of some key enzymes of the fatty acid β-oxidation pathway. Shown are mean relative expression values in CD44^+^CD117^+^ cells compared to CD44^+^CD117^−^ cells ± SD measured in ten samples. *p < 0.05. I. Unfractionated EOC effusion cells were incubated with the fatty acid β-oxidation pathway inhibitor Etomoxir, and the viability of CD44^+^CD117^+^ and CD44^+^CD117^−^ cells analysed at different times by Annexin V/PI staining. The graph shows mean cell viability ratios ± SD calculated from three consecutive experiments *p < 0.05.

Results of qRT-PCR revealed that the expression of the *GLUT1* transporter was comparable in the two populations, whereas the levels of hexokinase II (*HKII*), phosphofructokinase (*PFK*) and total pyruvate kinase (*PKM*) were significantly higher in CD44^+^CD117^+^ cells (Figure 2A). Key TCA/ETC enzymes, such as citrate synthase (*CS*), isocitrate dehydrogenase (*IDH2*), and *ATP5B*, were also more highly expressed in the CD44^+^CD117^+^ subset (Figure 2A). To validate these findings, we investigated the protein levels of some of the above enzymes. It is known that GLUT1 exerts its glucose transporter function only when exposed on the outer cell membrane [16]. Consistent with mRNA expression data, flow cytometry analysis of permeabilized cells indicated that the two subsets contained comparable total amounts of GLUT1 protein (Figure 2B, left panel). However, CD44^+^CD117^+^ cells expressed much higher levels of GLUT1 protein on the cell surface (Figure 2B, right panel). This finding was corroborated by analysis of fluorescent glucose uptake: CD44^+^CD117^+^ cells bound FITC-labelled glucose (2-NBDG) much more avidly, compared to CD44^+^CD117^−^ cells (Figure 2C). Results of Western blots (WB) verified that HKII and the TCA enzyme IDH2 were much more abundant in the CD44^+^CD117^+^ subset (Figure 2D).

We also tested the expression of two enzymes involved in PPP, glucose-6-phosphate dehydrogenase (*G6PD*) and ribulose-5-phosphate isomerase (*Ru5PI*). The mRNAs coding for these enzymes were significantly more abundant in CD44^+^CD117^+^ cells (Figure 2E). This observation was confirmed at the protein level for G6PD (Figure 2F). Accordingly, incubation of ascitic effusion cells with dehydroepiandrosterone (DHEA), a known PPP inhibitor [17], resulted in a significant decrease in the viability of CD44^+^CD117^+^ cells, whereas no effect was observed in the CD44^+^CD117^−^ subset (Figure 2G).

An analysis of enzymes involved in the fatty acid β-oxidation pathway, i.e. carnitine O-acetyltransferase (*CPT2*), enoyl-CoA hydratase (*ECHS1*), acetyl-CoA acyl-transferase 1 (*ACAA1*), ketoacyl-CoA thiolase 2 (*ACAA2*) and glycerol kinase [18], showed that all were more highly expressed at the mRNA level in CD44^+^CD117^+^ cells, compared to the single-positive counterpart (Figure 2H). The importance of this pathway in ovarian CSC was buttressed by an analysis of the effect of Etomoxir, an inhibitor of mitochondrial CPT1, which regulates the transport of long-chain fatty acids from the cytosol to mitochondria for β-oxidation [19]. After 6 hr incubation with Etomoxir, CD44^+^CD117^+^ cells displayed a significant reduction in viability (Figure 2I), whereas CD44^+^CD117^−^ cells did not show any change, even after 24 hr of exposure to the drug.

### Ovarian CSC overexpress key enzymes controlling the fuelling of pyruvate into the TCA cycle

**Figure 3:**
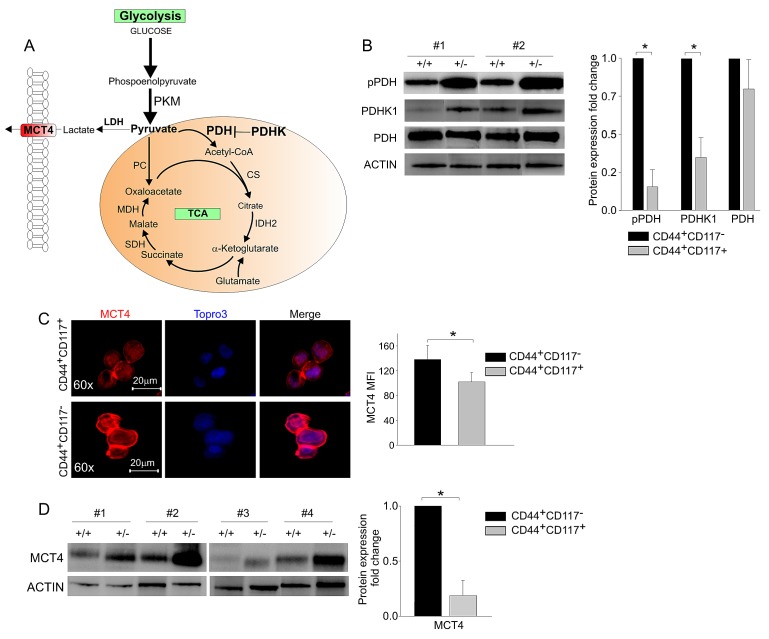
The key enzymes driving glucose commitment to the TCA cycle are highly expressed in CD44^+^CD117^+^ovarian cancer cells A. In this schematic representation of the major steps of glucose metabolism, the PKM enzyme catalyses the conversion of phosphoenolpyruvate to pyruvate, but the key enzyme that drives its ultimate destination to oxidative metabolism is pyruvate dehydrogenase (PDH). The action of PDH is in turn regulated by PDHK, which phosphorylates PDH, thus inhibiting its function. TCA, tricarboxylic acid. B. WB analysis of phospho-PDH (pPDH), PDHK1, and total PDH expression in FACS-sorted CD44^+^CD117^+^ (+/+) and CD44^+^CD117^−^ (+/−) cells from EOC effusions. Results in two representative samples are shown on the left. Ratios of protein expression in CD44^+^CD117^+^ cells *vs.* CD44^+^CD117^−^ cells were calculated as described in the legend to Fig. 2D. The graph shows mean expression ratios ± SD calculated from three experiments. *p < 0.05. C. FACS-sorted CD44^+^CD117^+^ and CD44^+^CD117^−^ cells from EOC ascitic fluid samples were analysed by confocal immunofluorescence for the expression of the MCT4 lactate transporter. Nuclei were stained with TOPRO3. One representative experiment is shown on the left, and the histogram on the right shows mean values ± SD of MCT4 mean fluorescence intensity (MFI) from 10 different fields. *p < 0.05. D. CD44^+^CD117^+^ (+/+) and CD44^+^CD117^−^ (+/−) cells from EOC ascitic fluid samples were FACS-sorted and MCT4 expression analysed by WB. Results from four representative samples are shown on the left. The ratio of MCT4 expression in CD44^+^CD117^+^ cells vs. CD44^+^CD117^−^ cells was calculated as described in the legend to Fig. 2D. The graph shows the mean expression ratio ± SD calculated from six experiments. *p < 0.05.

We next measured the expression levels of the key enzymes driving the commitment of pyruvate to the TCA cycle (Figure 3A). The ultimate destination of pyruvate to mitochondrial utilization is controlled by pyruvate dehydrogenase (PDH), whose activity is in turn regulated by PDH kinase (PDHK), which phosphorylates PDH, thus inactivating its function [20]. Interestingly, while equal levels of total PDH were found by WB in the two subsets (Figure 3B), the levels of PDHK1 and of the inactive form of PDH (phospho-PDH) were significantly lower in CD44^+^CD117^+^ cells (Figure 3B). These data strongly suggest that pyruvate fuelling into the TCA cycle is privileged in CD44^+^CD117^+^ cells. Consistent with these findings, immunofluorescence analysis of *ex vivo* isolated EOC cells revealed significantly higher expression of the lactate transporter MCT4 in CD44^+^CD117^−^ cells, compared to the CD44^+^CD117^+^ population (Figure 3C). This observation was confirmed by WB, which showed significantly lower levels of MCT4 in the CSC (Figure 3D). Altogether, these data suggest that pyruvate is preferentially conveyed to the TCA cycle in CD44^+^CD117^+^ cells, while the higher MCT4 expression found in the CD44^+^CD117^−^ population is consistent with a more pronounced lactate flux and Warburg-like phenotype of this latter subset.

A role for routing pyruvate into the TCA cycle has been advanced for PKM, which exists in several isoforms, and regulates conversion of phosphoenolpyruvate to pyruvate [21, 22]. Results of qRT-PCR to compare the relative proportion of *PKM1* and *PKM2* between CD44^+^CD117^+^ and CD44^+^CD117^−^ cells showed that the *PKM2* isoform prevailed in both subsets, but we did not observe any difference in the expression of the two isoforms between CSC and the non-stem counterpart (Figure S1).

### Ovarian CSC show higher mitochondrial activity and are more sensitive to ETC inhibitors

**Figure 4:**
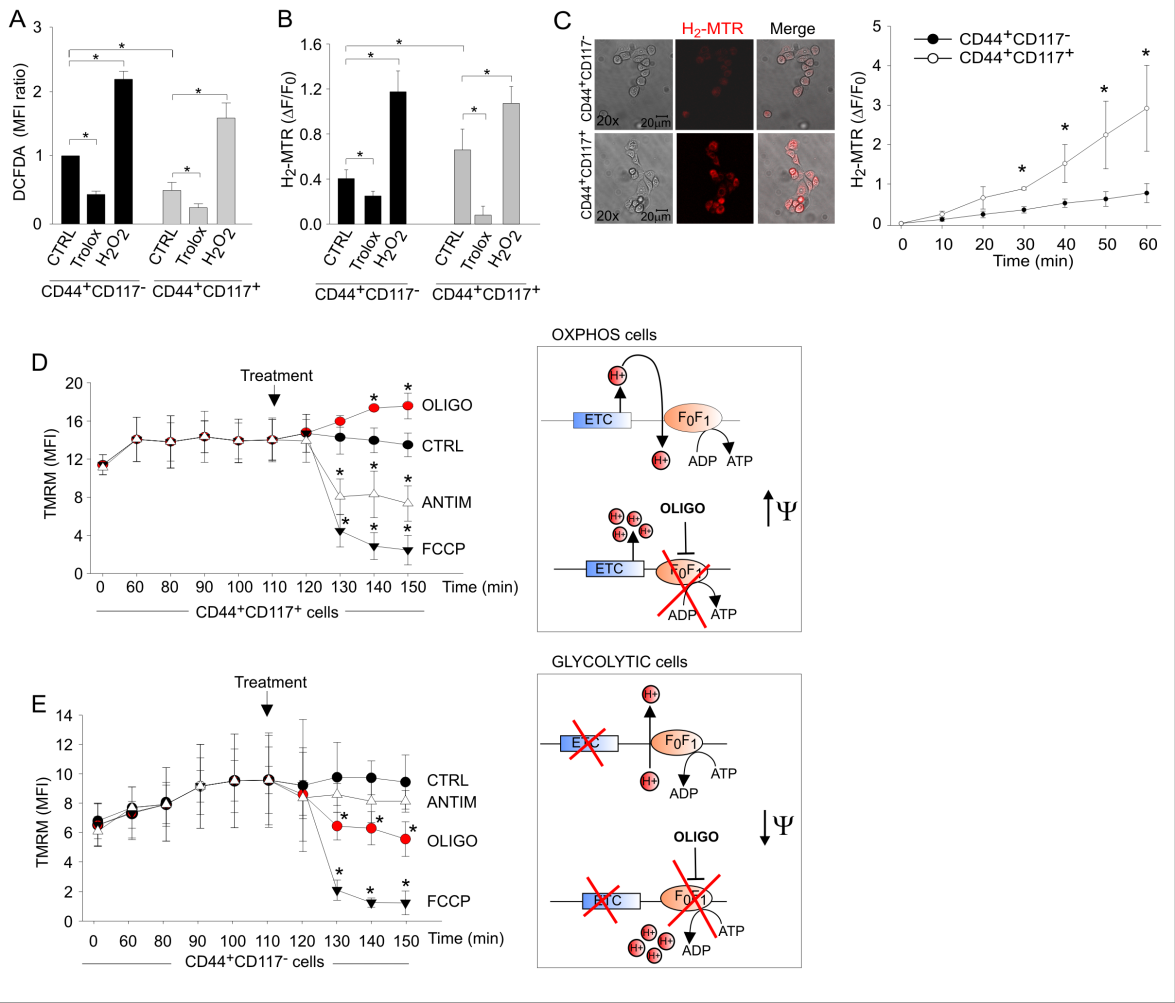
Ovarian cancer CD44^+^CD117^+^ cells exhibit higher mitochondrial ROS production and an OXPHOS metabolic profile compared to CD44^+^CD117^−^ cells A. Total ROS levels in unfractionated EOC effusion cells were measured by flow cytometry with the DCFDA probe following staining with anti-CD44 and anti-CD117 antibodies. Exogenous H_2_O_2_ and the ROS scavenger Trolox were used as positive and negative control, respectively. Mean Fluorescence Intensity (MFI) ratios were calculated dividing the MFIs measured for CD44^+^CD117^+^ cells by those obtained for CD44^+^CD117^−^ cells. The graph shows mean MFI ratios ± SD from ten experimental repeats. *p < 0.05. B. Mitochondrial ROS levels in CD44- and CD117-labelled EOC effusion cells were measured using the H_2_-MTR probe. Changes in H_2_-MTR fluorescence were measured by flow cytometry after 20 minutes of incubation. H_2_-MTR ΔF/F_0_ values were calculated as described in the *Methods*. Mean values ± SD from twelve experiments are shown. *p < 0.05. C. Time-lapse laser scanning microscopy of mitochondrial ROS production in FACS-sorted CD44^+^CD117^−^ and CD44^+^CD117^+^ populations. Images obtained at 60 min in a representative experiment are shown on the left; the graph shows mean values ± SD of H_2_-MTR ΔF/F_0_ recorded in at least 60 cells per time point per sample in 3 independent experiments. *p < 0.05. D, E. Mitochondrial membrane potential (Δψ_m_) in CD44^+^CD117^+^ (D) and CD44^+^CD117^−^ (E) cells was measured by flow cytometry with tetramethylrhodamine methyl ester (TMRM). FCCP was used as a control for the specificity of the probe, whereas antimycin and oligomycin were added to test the Δψ_m_-generating mode of F_1_F_O_-ATP synthase. The graphs show mean values ± SD of TMRM MFI values in five experimental repeats. CD44^+^CD117^+^ cells showed a significant hyperpolarization in response to oligomycin, thus demonstrating the usage of the F_1_F_O_-ATP synthase in a Δψ_m_-dissipating “OXPHOS” mode (D, right panel). In contrast, CD44^+^CD117^−^ cells showed a significant depolarization in response to oligomycin, thus demonstrating a “reverse” Δψ_m_-generating mode of function of the F_1_F_O_-ATP synthase, which is strongly suggestive of a glycolytic metabolic profile (right panel in E). In both cell populations, mitochondria were rapidly and comparably depolarized in response to the control protonophore FCCP. *p < 0.05 compared to control (TMRM).

We thus addressed the respiratory activity of the two populations, starting with a comparison of total and mitochondrial reactive oxygen species (ROS) production in living EOC effusion cells labelled with anti-CD44 and anti-CD117 antibodies. Total ROS levels, measured by flow cytometry of cells incubated with the H_2_O_2_-sensitive fluorescent probe DCFDA, were significantly higher in the non-CSC population than in the CD44^+^CD117^+^ subset (Figure 4A). Mitochondrial ROS levels were measured using MitoTracker Red (H_2_-MTR), a mitochondria-specific probe that is converted to fluorescent MTR upon oxidation by H_2_O_2_. Changes in H_2_-MTR fluorescence were measured using two approaches, (i) flow cytometry of unfractionated EOC cells following staining with anti-CD44/CD117 antibodies and (ii) *in situ* measurement by time-lapse laser scanning microscopy. Results showed that both the levels (Figure 4B) and rate of accumulation (Figure 4C) of mitochondrial ROS were significantly higher in the CD44^+^CD117^+^ than in the CD44^+^CD117^−^ cells.

To determine the metabolic *status* of the cells, independent of mitochondrial mass (Figure S2) [23], we investigated the effects of oligomycin on mitochondrial membrane potential (Δψ_m_). This mitochondrial F_1_F_O_-ATP synthase inhibitor can be employed to discriminate whether the synthase is used to dissipate Δψ_m_ in order to produce ATP (as expected in cells using OXPHOS), or rather functions in a “reverse mode” to maintain Δψ_m_ at the expenses of ATP hydrolysis (as expected in cells diverting pyruvate from TCA cycle) [24, 25]. Changes in Δψ_m_ were monitored using tetramethylrhodamine methyl-ester (TMRM), a fluorescent probe that accumulates in mitochondria in a Δψ_m_-dependent manner. CD44^+^CD117^+^ cells showed a significant hyperpolarization in response to oligomycin, thus demonstrating that in these cells the F_1_F_O_-ATP synthase works in a Δψ_m_-dissipating “OXPHOS” mode (Figure 4D). In contrast, CD44^+^CD117^−^ cells showed a significant depolarization in response to oligomycin, thus indicating a “reverse” mode of function of the F_1_F_O_-ATP synthase, which is strongly suggestive of a glycolytic, Warburg-like profile (Figure 4E). Upon addition of antimycin (which inhibits the Complex III of ETC), CD44^+^CD117^+^ cells showed a significant depolarization (Fig. 4D), thus implying that the ETC was the main Δψ_m_-generating pathway in these cells. On the contrary, antimycin did not alter Δψ_m_ in the CD44^+^CD117^−^ population (Figure 4E), consistent with poor ETC fuelling in these cells. Both cell subsets showed a comparable mitochondrial depolarization (Figure 4D-E) upon treatment with the control protonophore carbonylcyanide-*p*-trifluoromethoxyphenyl hydrazone (FCCP).

**Figure 5:**
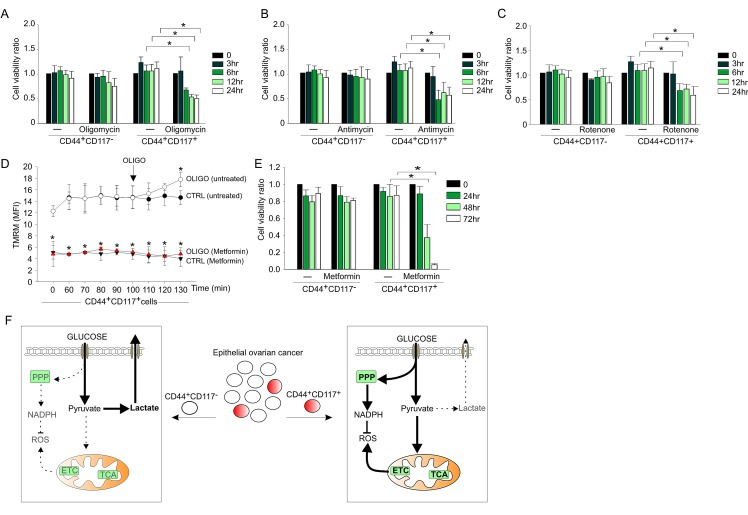
OXPHOS inhibitors significantly affect viability of CD44^+^CD117^+^ but not CD44^+^CD117^−^ovarian cancer cells A-C. Unfractionated EOC effusion cells were incubated in the absence or presence of oligomycin (A), antimycin (B) or rotenone (C), and the viability of CD44^+^CD117^+^ and CD44^+^CD117^−^ cells was analysed at different times by Annexin V/PI staining. Cell viability ratios were calculated as described in the *Supplemental Data*. Shown are mean values ± SD from three consecutive experiments. *p < 0.05. D. To validate the effect of metformin on ETC, mitochondrial membrane potential (Δψ_m_) was measured by flow cytometry with the TMRM probe in CD44^+^CD117^+^ cells from EOC effusions, either untreated (ctrl) or after 90 min of treatment with 1 mM metformin. Oligomycin was added to test the Δψ_m_-generating mode of F_1_F_O_-ATP synthase (see Fig. 4E). Shown are mean MFI values ± SD from five experimental repeats. *p < 0.05. E. Unfractionated EOC effusion cells were incubated in the absence or presence of metformin (1 mM) and cell viability evaluated in CD44^+^CD117^+^ and CD44^+^CD117^−^ cells at various time intervals. Cell viability ratios were calculated as described in the *Supplemental Data,* with results expressed as mean values ± SD from three consecutive experiments. *p < 0.05. F. Schematic model of the metabolic profile of CD44^+^CD117^+^ (CSC) and CD44^+^CD117^−^ (non-CSC) cells from EOC ascitic effusions. Left panel: bulk tumor cells present a glycolytic metabolic profile, in which most of the glucose entering the cells is converted to pyruvate, and eventually extruded as lactate by the MCT4 transporter. Right panel: in the CSC subset, a large proportion of the glucose entering the cell is converted to pyruvate to fuel the TCA cycle and ETC, which in turn increases mitochondrial ROS production. In these cells, a significant glucose fraction is shunted to PPP to potentiate the redox power of the cells (through NADPH and ROS scavenger generation). The thick arrows denote more active pathways, and the dotted arrows indicate non-preferential pathways. ETC, electron transport chain; PPP, pentose phosphate pathway; ROS, reactive oxygen species; TCA, tricarboxylic acid.

To corroborate these data, we compared the effects of the ETC inhibitors oligomycin, rotenone (an inhibitor of Complex I) and antimycin on the viability of the CD44^+^CD117^+^ and CD44^+^CD117^­-^ cells. Inhibition of oxidative phosphorylation had a dramatic effect on the survival of CD44^+^CD117^+^ cells, with a >50% decrease in viability after 6 hr treatment (Figure 5,), whereas CD44^+^CD117^−^ cells did not show any change in viability when cultured for 24 hours in the presence of each inhibitor. Similar results were obtained with metformin, which also inhibits mitochondrial Complex I and is now in the spotlight as a promising anticancer drug [26]. In accordance with an anti-Complex I effect, the mitochondrial membrane potential of CD44^+^CD117^+^ cells was dramatically reduced following 90 min incubation with metformin (Figure 5D), and, as expected, the addition of oligomycin did not increase Δψ_m_, a response that was instead evident in the control population. Cultivation of the cells in the presence of metformin resulted in a dramatic decrease in the viability of the CD44^+^CD117^+^ population, whereas no change was recorded in the CD117^−^ counterpart (Figure 5E). A tentative model of the metabolic profile of CD44^+^CD117^+^ *vs*. CD44^+^CD117^−^ EOC effusion cells that draws from these findings is proposed in Figure 5F.

### Ovarian CSC resist *in vitro* and *in vivo* glucose deprivation, while maintaining their CSC properties

**Figure 6:**
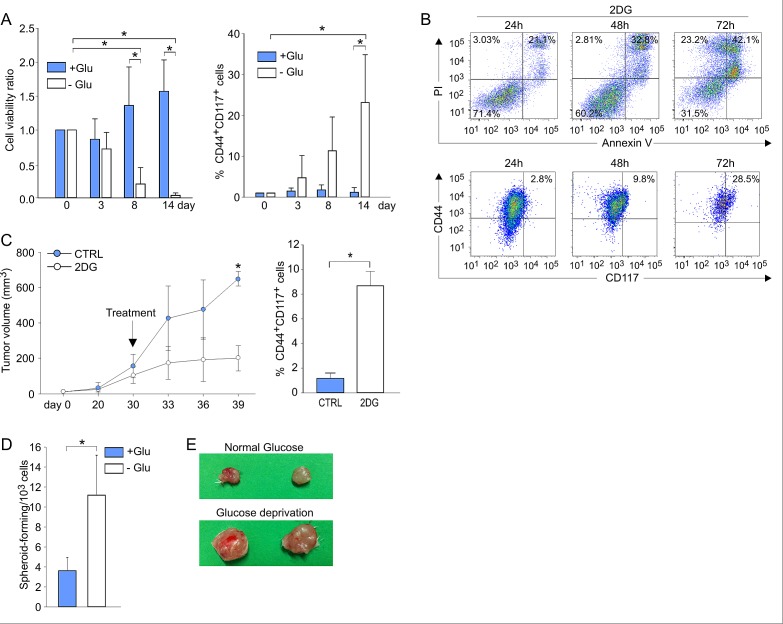
Ovarian cancer CD44^+^CD117^+^ cells resist *in vitro* and *in vivo* glucose deprivation, while maintaining their CSC properties A. Unfractionated EOC ascitic effusion cells were cultured in the presence (+Glu) or absence (-Glu) of glucose for 14 days; cell viability (left panel) and the percentage of CD44^+^CD117^+^ cells (right panel) were evaluated at different time points. Shown are mean values ± SD from ten consecutive experiments. *p < 0.05. B. Flow cytometry analysis of cell viability (upper panel) and CD44/CD117 co-expression (lower panel) of unfractionated EOC effusion cells cultured in the presence of the glucose analogue 2-DG. One representative experiment out of three is shown. C. Six RAG-2 γ^−/−^ mice were injected with 5 × 10^5^ cells/flank from EOC effusion xenografts; when the tumors reached a volume of 100 mm^3^, 3 animals were treated with 2-DG, and 3 received saline as a control (CTRL). The left panel shows the kinetics of tumor growth, and the right panel reports the percentage of CD44^+^CD117^+^ cells in the resulting tumor masses. Data are mean values ± SD for six tumors/group. *p < 0.05 D. unfractionated EOC effusion cells were cultured in the presence (+Glu) or absence (-Glu) of glucose. After 14 days the ability to generate spheroids was evaluated by ELDA. Shown are the mean values of spheroid-forming precursors/10^3^ cells ± SD in five consecutive experiments. *p < 0.05. E. RAG-2 γ^−/−^ mice were injected with 1 × 10^5^ cells/flank from EOC effusion xenografts. Before inoculation, the cells were cultured for two weeks in standard medium (upper panel) or in medium without glucose (lower panel).

The finding of high glucose uptake and elevated PPP and OXPHOS activity in the CSC prompted us to investigate their response to glucose deprivation. This issue was particularly compelling in view of data indicating that anti-angiogenic therapy causes glucose starvation in experimental tumors [27] and is associated with enhanced survival of CSC in patients [28, 29]. We thus cultured ascitic effusion cells in the presence and absence of glucose, and evaluated their phenotypic and functional profiles. Glucose starvation of EOC effusion cells was associated with a dramatic decrease in viability (Figure 6A, left panel), due to the preferential death of CD44^+^CD117^−^ cells (not shown), and a significant enrichment in CD44^+^CD117^+^ cells (Figure 6A, right panel), conditions reverted by the re-addition of glucose (Figure S3). Cultivation of EOC effusion cells in the presence of 2-DG, a glucose analogue which prevents glucose accumulation *via* HKII blockade [30], resulted in a dramatic decrease in cell viability, while the percentage of CD44/CD117 co-expressing cells increased in parallel (Figure 6B). Interestingly, administration of 2-DG to mice injected with ovarian cancer xenografts caused a reduction in the tumor growth rate (Figure 6C, left panel). Intriguingly, however, a significant enrichment in CD44^+^CD117^+^ cells was found in tumors from 2-DG-treated animals, compared to controls (Figure 6C, right panel).

Nevertheless, glucose-starved cells fully maintained their original CSC properties. In fact, as judged by ELDA, culture in the absence of glucose was associated with a significantly higher ability to form spheroids, compared to cells kept under standard conditions (Figure 6D). Furthermore, 4/4 RAG-2 γ^−/−^ mice injected i.p. with 1 × 10^3^ cells previously cultured in the absence of glucose developed tumors, whereas no tumor formation was observed in 6 animals injected with the same number of cells kept under normal culture conditions (not shown). In addition, tumors obtained by s.c. injection of 1 × 10^5^ glucose-starved or non-starved cells were much larger when glucose-starved cells were injected (Figure 6E). Altogether, these data indicate that glucose-starved CD44^+^CD117^+^ cells maintain the fundamental properties of CSC, and that glucose deprivation resistance is a consistent feature of the CD44^+^CD117^+^ subset in different *in vitro* and *in vivo* conditions.

### Glucose deprivation modulates the metabolic profile of ovarian CSC, while sparing their OXPHOS profile

**Figure 7:**
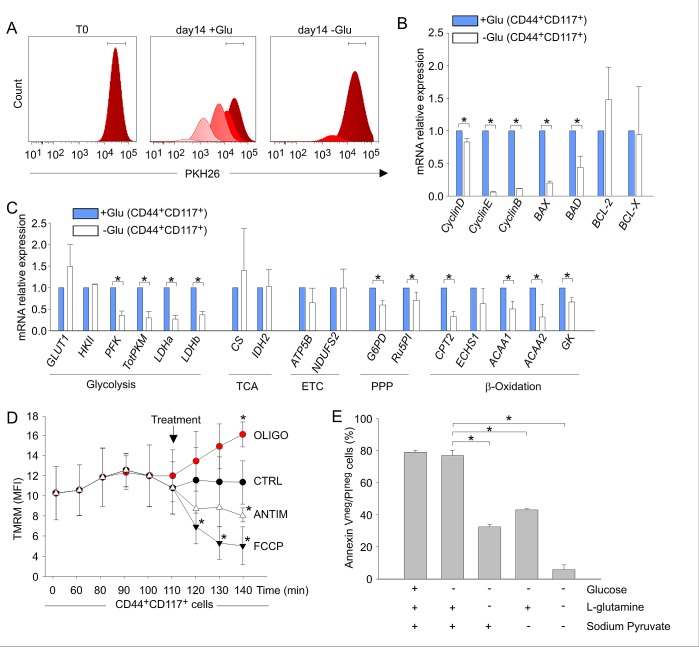
Glucose deprivation modulates the metabolic profile of ovarian cancer CD44^+^CD117^+^ cells, while sparing their OXPHOS profile A Unfractionated EOC effusion cells were labelled with PKH26, and maintained in culture in the presence (+Glu) or absence (-Glu) of glucose. PKH26 staining was evaluated in the two culture conditions 14 days later, and analysed by FlowJo to monitor cell division. One representative experiment out of four is shown. B. Unfractionated EOC effusion cells were cultured in the presence (+Glu) or absence (-Glu) of glucose, and the expression of cyclins and apoptosis-associated genes was evaluated by qRT-PCR 14 days later in FACS-sorted CD44^+^CD117^+^ cells. The relative expression of each mRNA in glucose-starved CD44^+^CD117^+^ cells compared to control CD44^+^CD117^+^ cells was calculated as described in the *Supplemental Data*. Mean values ± SD from six samples are shown. *p < 0.05. C. The expression of a panel of metabolism-associated genes was evaluated in CD44^+^CD117^+^ cells cultured as described in B. Relative mRNA expression values were calculated as described above. Shown are mean values ± SD from six samples. *p < 0.05. D. Mitochondrial membrane potential (Δψ_m_) was measured by flow cytometry as detailed in Figure 4D-E in CD44^+^CD117^+^ cells previously cultured for 14 days in the absence of glucose. Antimycin and oligomycin were added to test the Δψ_m_-generating mode of F_1_F_O_-ATP synthase. Glucose-starved CD44^+^CD117^+^ cells showed a profile fully comparable to that of non-starved CSC (see Figure 4D), with a significant hyperpolarization in response to oligomycin, demonstrating that glucose deprivation did not change the Δψ_m_-dissipating “OXPHOS” mode of the F_1_F_O_-ATP synthase. Shown are mean TMRM MFI values ± SD from three experiments. *p < 0.05 compared to control. E. Unfractionated EOC effusion cells were cultured for 14 days in the absence of glucose, and then transferred to media with/without glucose, L-glutamine and sodium pyruvate, as specified on the abscissa. Cell viability was analysed by Annexin V/PI staining 72 h later. Shown are mean percentages of live cells (Annexin V^neg^/PI^neg^) ± SD from three experiments. *p < 0.05.

The relative increase in CD44^+^CD117^+^ cells in the absence of glucose was not due to their *in vitro* expansion or *de novo* co-expression of these receptors. In fact, the absolute number of input CD44^+^CD117^+^ cells did not significantly change over the glucose starvation period (Table S2). Moreover, when ascitic effusion cells were labelled with PKH26, a fluorescent dye used to monitor cell division [31], mostly PKH^high^ cells were found after glucose deprivation, whereas in normal culture conditions several peaks of PKH intensity were observed, reflecting serial dilution of the dye due to cell replication (Figure 7A). These findings indicated that CD44^+^CD117^+^ cells did not undergo proliferation, but remained quiescent when cultured in the absence of glucose. This observation was corroborated by qRT-PCR analysis of *cyclin D*, *E*, and *B* expression, which was strongly down-modulated in glucose-starved cells (Figure 7B). Interestingly, expression of the pro-apoptotic genes *BAX* and *BAD* was also significantly reduced in glucose-starved CD44^+^CD117^+^ cells, whereas no change in expression of the anti-apoptotic genes *BCL-2* and *BCL-X* was observed (Figure 7B).

Glucose-starved CD44^+^CD117^+^ cells showed an overall down-regulation in expression of glucose metabolism-related genes, as well as in genes involved in fatty acid β-oxidation, compared to cells cultured in the presence of glucose (Figure 7C). However, *GLUT1* was expectedly up-regulated by glucose deprivation (Figure 7C); interestingly, the TCA/ETC genes *CS*, *IDH2* and *ATP5B* did not change significantly following culture in the absence of glucose (Figure 7C). Accordingly, the response of mitochondrial Δψ_m_ to oligomycin showed a comparable OXPHOS profile in freshly isolated (see Figure 4D) and glucose-starved CD44^+^CD117^+^ cells (Figure 7D). Thus, it is reasonable to conclude that ovarian CSC resist glucose starvation by entering a quiescent state, while maintaining their preferential OXPHOS profile. It is possible that ovarian CSC could resist glucose deprivation thanks to the exploitation of anaplerotic pathways such as glutaminolysis to support ATP production. To test this hypothesis, we cultured unfractionated EOC cells under glucose deprivation for 14 days, followed by an additional L-glutamine and/or sodium pyruvate starvation for 72 hr. As shown in Figure 7E,-glutamine deprivation of glucose-starved CD44^+^CD117^+^ cells caused a >50% decrease in cell viability, while withdrawal of both L-glutamine and sodium pyruvate caused death of virtually all the cells (Figure 7E).

## DISCUSSION

In this study we investigated the metabolic profile of CSC obtained *ex vivo* from ascitic effusions of EOC-bearing patients. CSC biology is intricate [5], and the CSC phenotype may encompass a gradient, where tumor progenitors at different stemness levels are comprised under the umbrella of “stem” marker expression [32]. Nonetheless, our data point to the ovarian CD44^+^CD117^+^ subset as a *bona fide* CSC population, which clearly differs molecularly and functionally from the bulk of differentiated tumor cells. In fact: i) ovarian CD44^+^CD117^+^ cells overexpress key genes associated with glucose uptake, ETC/TCA, PPP, and fatty acid β-oxidation; ii) CD44^+^CD117^+^ cells have lower levels of PDHK1 and phospho-PDH than CD44^+^CD117^−^ cells, indicating their propensity to direct pyruvate utilization towards the Krebs cycle; iii) CD44^+^CD117^+^ cells produce higher levels of mitochondrial ROS, and die when the mitochondrial respiratory chain is blocked. Moreover, a clear-cut qualitative difference in response to inhibition of the F_1_F_0_-ATP synthase by oligomycin demonstrates that in CD44^+^CD117^+^ cells this enzyme is used to dissipate Δψ_m_ in order to produce ATP, whereas in CD44^+^CD117^−^ cells it functions in a “reverse mode” to maintain Δψ_m_ at the expenses of ATP hydrolysis.

Although we could not directly prove higher oxygen consumption and lower lactate production in CSC, as FACS sorting-associated stress precludes determining these functional parameters (Figure S4), the finding that CD44^+^CD117^+^ and CD44^+^CD117^−^ cells express different amounts of PDHK1/phospho-PDH is a crucial clue indicating that ovarian CSC preferentially exploit the mitochondrial respiratory pathway compared to the non-CSC counterpart. Thus, it is tempting to speculate that ovarian CSC escape the Warburg effect. On the contrary, the non-stem counterpart exhibits a metabolic profile compatible with aerobic glycolysis; indeed, our ascitic effusion samples conceivably do not undergo hypoxia and eventual HIF-1 activation. This observation may recall what has been reported in senescence, where cells undergo a pseudo-hypoxic state in the absence of reduced oxygen tension, mostly driven by mTOR [33]. In a sense, single-positive EOC cells look like cells undergoing “chronological senescence” [33, 34], as suggested by a sustained Warburg effect and lack of OXPHOS respiration in the absence of hypoxia [35]. On the contrary, ovarian CSC show intact respiratory activity, suggesting a non-senescent phenotype, which is conceivably essential for the maintenance of a self-renewing reservoir.

It has been reported that the balance between the *PKM1* and *PKM2* isoforms of *PKM* may be critical in directing pyruvate to either lactate production or mitochondrial utilization [21, 36]. Indeed, in models of enforced expression or silencing of these enzymes in malignant cell lines, PKM2 expression is associated with the Warburg effect [37, 38]. Although we observed a significant elevation in total *PKM* in the CSC subset, we did not observe any difference in the relative proportion of *PKM1* and *PKM2* between CD44^+^CD117^+^ and CD44^+^CD117^−^ cells. However, molecular and biochemical data on PKM2 may be misleading and may not reflect its actual enzymatic activity, as PKM2 function depends on the formation of dimers/tetramers [21], detection of which is possible under controlled conditions in established cell lines, but unfeasible in our setting.

Our data clearly indicate that EOC CD44^+^CD117^+^ cells are much less dependent on glucose than the CD44^+^CD117^−^ population. This finding is in line with recent observations on *in vitro* resistance of glioblastoma CSC to glucose deprivation [39]. Intriguingly, although the metabolic profile of their CD133^+^ population was not characterized, these investigators observed pronounced expression of the glucose transporter GLUT3. Our finding of strong surface expression of GLUT1 in ovarian CSC leads us to ask why these relatively quiescent OXPHOS-driven cells should manifest high glucose avidity compared to the bulk tumor cells, and how limiting the Warburg effect might be advantageous to ovarian CSC. We found that ovarian CSC are characterized by high PPP activity, a finding consistent with their high glucose uptake and HKII expression. One key function of the PPP is to maintain high levels of NADPH, which in turn is essential for recharging ROS-scavenging enzymes. Thus, the finding of higher PPP activity, along with high OXPHOS and mitochondrial ROS production, might point to differences in ROS homeostasis between CSC and the bulk tumor cell population, in order to preserve the integrity of the former, and avoid further DNA damage or irreversible opening of mitochondrial pores [40]. In addition, the higher expression of fatty acid β-oxidation enzymes by CD44^+^CD117^+^ cells could indicate that these metabolites also play an important role within the CSC energy economy, probably to produce intermediates for the Krebs cycle. In this regard, intriguing results obtained in a different setting have shown that inhibition of fatty acid β-oxidation by Etomoxir/Orlistat is associated with selective pro-apoptotic effects in human acute myeloid leukemia progenitors [41].

The metabolic profile and glucose deprivation resistance of ovarian CSC could also help in explaining the refractoriness of EOC to therapies restricting oxygen and nutrient supply. Studies of experimental tumors [42] and human cancer [28, 29, 43] showed that anti-angiogenic therapies are associated with an increase in the percentage of CSC in the residual tumor mass; indeed, our data on the effect of *in vivo* 2-DG treatment on tumor generation further reinforce this idea. We observed that CSC undergo complete quiescence in the absence of glucose, and down-regulate most metabolic activities, while maintaining an OXPHOS profile; this phenomenon could be instrumental in helping CSC to escape damage in hypo-oxygenated tumor areas, but the mechanisms determining this phenotype are unclear. In this regard, the observed activity of metformin on CD44^+^CD117^+^ cells, which confirms and extends previously reported effects on pancreatic CSC [44], deserves further investigation, and suggests that approaches combining anti-angiogenic drugs and metformin could be effective for eradicating CSC.

## METHODS

### Primary samples and *in vitro* culture

Ascitic fluid and tumor samples of EOC patients were obtained following informed consent from 45 patients. Cells were maintained in RPMI-1640 medium supplemented with 10% FBS (GIBCO Invitrogen, Monza, Italy), 1% sodium pyruvate (Lonza, Basel, Switzerland), and 1% L-glutamine (GIBCO). Cells were cultured at 37°C, 5% CO_2_, and harvested at confluence using trypsin-EDTA (Invitrogen). In a set of experiments, the cells were cultured in the absence of glucose; at the end of the starvation period, glucose was added and marker expression evaluated 10 days later. In another set of experiments, the cells were cultured for 14 days in the absence of glucose, and then transferred to medium without glucose, without glucose and L-glutamine, or without glucose, L-glutamine and sodium pyruvate. Cell viability was evaluated 72 hr later by flow cytometry with Annexin V/PI staining, as detailed in *Supplemental Data*.

### Flow cytometry

The cells were stained with Live-Dead (Pacific Blue, 1:600; Invitrogen) to discriminate living cells. The following anti-human monoclonal antibodies were used: anti-CD44 (1:1,000; Abcam, Cambridge, U.K.), anti-CD117 (non-activating AC126 clone, 1:10; Miltenyi Biotec, Bergish Gladbach, Germany), anti-CD45 (1:10; Miltenyi Biotec), anti-CK7 (1:25; Abcam), anti-GLUT1 (1:1,000; Abcam). Intracellular staining was performed after fixation with 4% paraformaldeyde and permeabilization with 0.1% Triton X-100. After incubation with unconjugated antibodies, the cells were incubated for 30 min with the appropriate secondary antibody (Alexa 1:500; Invitrogen). All the cytofluorimetric analyses were performed using a FACS LSRII (BD Bioscience, Franklin Lakes, NJ); data were collected from at least 1 × 10^5^ cells/sample and elaborated with FlowJo software (TreeStar, Ashland, OR). For FACS-sorting, antibody-labelled cells were separated with a MoFlo Astrios Cell Sorter (Beckman Coulter, Brea, CA); the purity of the sorted populations always exceeded 90%.

To evaluate glucose uptake, EOC effusion cells were labelled with anti-CD44 and anti-CD117 antibodies; 2-NBGD-FITC glucose (12.5 μM; Invitrogen) was then added and fluorescence intensity measured after 1 min. Cell viability was evaluated by incubating the cells for 15 min at 37°C with AnnexinV/PI staining kit (Roche, Basel, Switzerland); unless otherwise specified, results were expressed as the ratio between the percentage of Annexin V^neg^/PI^neg^ cells at the experimental time points and the percentage at time 0.

To evaluate the effect of glucose starvation on cell proliferation, EOC effusion cells were stained with PKH26 (Sigma Aldrich, St. Louis, MO) as described elsewhere [31] and seeded at 2x10^5^ cells/well in RPMI medium or RPMI without glucose (Sigma Aldrich), both supplemented as above. Flow cytometry analysis was performed 14 days later.

### Total and mitochondrial ROS production, membrane potential analysis and *in vitro* inhibition of metabolic enzyme activity

Unfractionated EOC effusion cells were labelled with anti-CD44 and anti-CD117 antibodies. For total ROS evaluation, the DCFDA probe (1 μM; Invitrogen) was added for 30 min at 37°C; cells were then washed and mean fluorescence intensity (MFI) values were recorded. For mitochondrial ROS evaluation, H_2_-MitoTracker Red probe (H_2_-MTR, 20 nM; BD Bioscience) was added to the cells at 37°C for 15 min; to verify the specificity of the assay for ROS produced by the ETC, we added pargyline, an inhibitor of mono-aminooxidases, which are an additional source of mitochondrial ROS [45]. H_2_O_2_ (100 μM) and Trolox (100 μM; both from Sigma Aldrich) were used as positive and negative controls. The kinetics of mitochondrial ROS production was calculated using the ΔF/F_0_ formula, where F_0_ is the fluorescent signal measured at time 0, and ΔF is the difference in MFI measured at the indicated time points minus the F_0_ value. Mitochondrial potential (Δψ_m_) was measured by incubating the cells with TMRM (20 nM; Invitrogen) in the presence of cyclosporin A (1.6 μM; Sigma Aldrich) for 20 min at 37°C. FCCP (100 nM; Sigma Aldrich) was used to induce depolarization; oligomycin (1 μM; Sigma Aldrich) and antimycin (1 μM; Sigma Aldrich) were added to perturb membrane potential.

To evaluate cell dependence on the ETC, fatty acid β-oxidation and the PPP, 1 × 10^6^ cells were plated in serum-free RPMI supplemented with oligomycin (1 μM), antimycin (1 μM), rotenone (1 μM), etomoxir (1 μM), DHEA (100 μM) or metformin (1 mM; all from Sigma Aldrich). Cell viability was evaluated with Annexin V/PI staining at different time points.

### RNA extraction, reverse transcription and gene card analysis

Please see *Supplemental Data.*

### Confocal and fluorescence microscopy analysis

For studying the kinetics of mitochondrial ROS production, FACS-sorted CD44^+^CD117^+^ and CD44^+^CD117^−^ cells were maintained at 37°C in DMEM medium (Sigma Aldrich) supplemented with 10% FBS, 1% sodium pyruvate, and 1% Hepes (Lonza). Cultures were pretreated with 2 mM pargyline, and then labeled with 20 nM H_2_-MTR. ROS production was measured using an LSM510 confocal laser microscope (Zeiss, Jena, Germany) equipped with a 37°C, 5% CO_2_ incubator using Helium Neon (543 nm) and Argon (488 nm) lasers. Laser intensity, pinhole aperture, and photomultiplier parameters were standardized to allow comparison of signals obtained in different samples; images were recorded at 1 min intervals for 60 min. The mean fluorescent signal of H_2_-MTR in mitochondria of individual cells was quantitated with the Zeiss Histogram software tool, and expressed as ΔF/F_0_ as above. To evaluate MCT4 expression, FACS-sorted CD44^+^CD117^+^ and CD44^+^CD117^−^ cells were stained with anti-MCT4 antibody (1:100; Santa Cruz Biotechnology, Dallas, TX); nuclei were stained with TOPRO3 (1:10,000; Invitrogen), and images recorded by confocal microscopy.

### Statistical Analysis

Data from replicate experiments were shown as mean values ± Standard Deviation [46]. Comparisons between groups were done by the two-tail Student's *t*-test and Mann-Whitney test, as appropriate.

## SUPPLEMENTARY MATERIAL


