# Supplementary material for: Cancer stem cells from epithelial ovarian cancer patients privilege oxidative phosphorylation, and resist glucose deprivation
Source: Oncotarget. 2014 May 26;5(12):4305–19. doi: 10.18632/oncotarget.2010 (PMC4147325; doi:10.18632/oncotarget.2010)
Supplement: Supplementary file 1 [file oncotarget-05-4305-s001.pdf]

Cancer stem cells from epithelial ovarian cancer patients privilege oxidative phosphorylation, and resist glucose deprivation

Supplementary Material

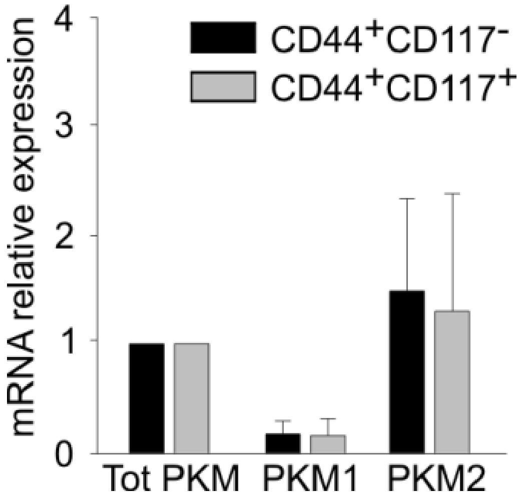

Figure S1:The expression of the PKM1 and PKM2 isoforms does not differ between $CD44^{+}CD117^{+}$ and $CD44^{+}CD117^{-}$ ovarian cancer populations

FACS-sorted $CD44^{+}CD117^{+}$ and $CD44^{+}CD117^{-}$ cells from EOC ascitic effusions were analyzed by qRT-PCR for the expression of total *PKM* and the *PKM1* and *PKM2* isoforms. The results were normalized to the housekeeping gene β_2-microglobulin (as described in *Supplemental Methods*). For each cell subset, mRNA expression of *PKM1* and *PKM2* was expressed related to total *PKM*, which was defined equal to 1. Data are expressed as mean value ± SD of ten different experiments.

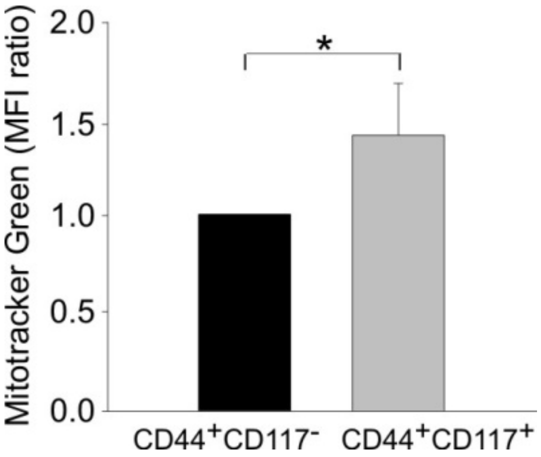

Figure S2:EOC CD44^{+}CD117^{+} cells show higher mitochondrial mass

Mitochondrial mass in CD44^{+}CD117^{-} and CD44^{+}CD117^{+} populations from EOC effusion cells was assessed by flow cytometry with the mitochondrial probe MitoTracker Green. Data represent mean values ± SD of MFI ratios in five consecutive experiments. *$p < 0.05$.

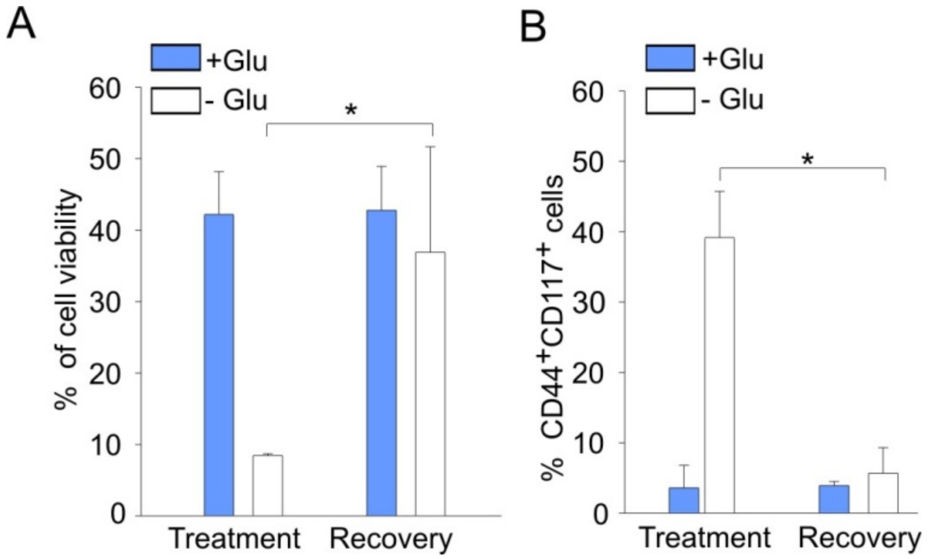

Figure S3:Glucose starved CD44$^+$CD117$^+$ cells reconstitute the entire cell population after glucose re-addition

A, B. Flow cytometry analysis of cell viability (A) and CD44/CD117 co-expression (B) of unfractionated EOC effusion cells cultured for 14 days in the presence (+Glu) or in the absence (-Glu) of glucose (Treatment) and after 10 days of glucose restoration (Recovery). Data are expressed as mean percent values ± SD of five different experiments. *$p < 0.05$.

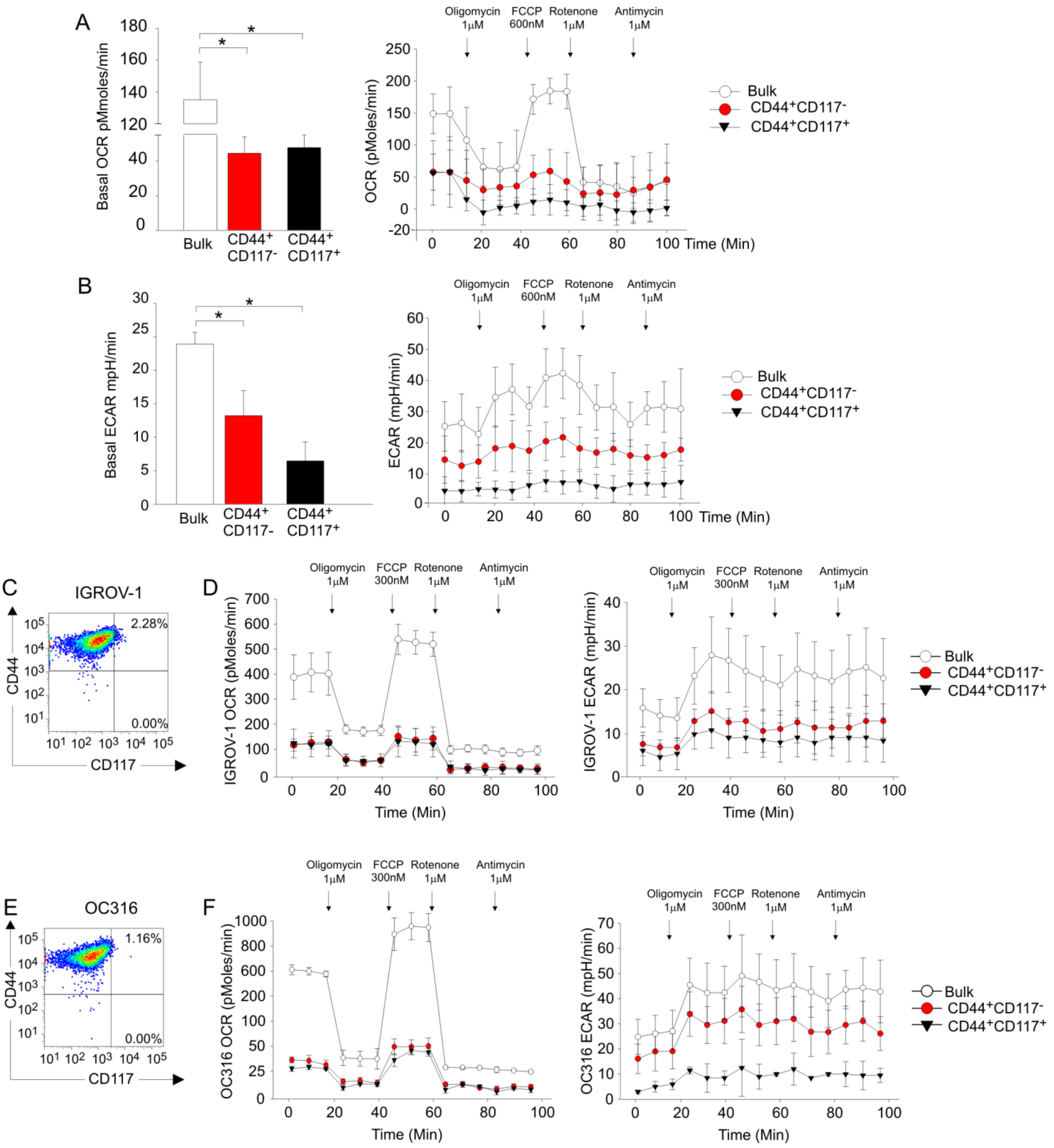
A
Basal OCR pMmoles/min
Bulk
CD44+ CD117-
CD44+ CD117+
Oligomycin 1μM
FCCP 600nM
Rotenone 1μM
Antimycin 1μM
OCR (pMoles/min)
Time (Min)
Bulk
CD44+CD117-
CD44+CD117+
B
Basal ECAR mpH/min
ECAR (mpH/min)
C
IGROV-1
2.28%
0.00%
CD44
CD117
D
FCCP 300nM
IGROV-1 OCR (pMoles/min)
IGROV-1 ECAR (mpH/min)
E
OC316
1.16%
0.00%
F
OC316 OCR (pMoles/min)
OC316 ECAR (mpH/min)

Figure S4:SeaHorse analysis of Oxygen Consumption Rate (OCR) and Extra-Cellular Acidification Rate (ECAR) in CSC-like and non-CSC-like cell subsets is heavily affected by FACS sorting procedures

A. Basal OCR in unsorted (bulk) and FACS-sorted $CD44^{+}CD117^{-}$ and $CD44^{+}CD117^{+}$ populations from EOC effusion samples (left panel). Data are expressed as mean values ± SD of ten different experiments. The OCR curves of bulk and sorted populations in a representative sample are shown in the right panel. The first three points of the graph indicate the basal OCR ratio. At different time points the following mitochondrial inhibitors were added: oligomycin (1 μM), FCCP (0.6 μM), rotenone (1 μM) and antimycin (1μM). *$p < 0.05$.

B. Basal ECAR in unsorted (bulk) and FACS-sorted $CD44^{+}CD117^{-}$ and $CD44^{+}CD117^{+}$ populations from EOC effusion samples (left panel). Data are expressed as mean values ± SD of ten different experiments. The ECAR curves of bulk and sorted populations in a representative sample are shown in the right panel. The first three points of the graph indicate the basal ECAR ratio. At different time points the following mitochondrial inhibitors were added: oligomycin (1 μM), FCCP (0.6 μM), rotenone (1 μM) and antimycin (1μM). *$p < 0.05$.

C. Representative flow cytometry analysis of CD44/CD117 co-expression in the ovarian cancer cell line IGROV-1.

D. OCR (left) and ECAR (right) curves of unsorted (bulk) and FACS-sorted $CD44^{+}CD117^{-}$ and $CD44^{+}CD117^{+}$ populations from the ovarian cancer cell line IGROV-1. The first three points of the graphs indicate the basal OCR and ECAR ratio. At different time points mitochondrial inhibitors were added as in **A** and **B**. One representative sample out of four is shown.

E. Representative flow cytometry analysis of CD44/CD117 co-expression in the ovarian cancer cell line OC316.

F. OCR (left) and ECAR (right) curves of unsorted (bulk) and FACS-sorted $CD44^{+}CD117^{-}$ and $CD44^{+}CD117^{+}$ populations from the ovarian cancer cell line OC316. The first three points of the graphs indicate the basal OCR and ECAR ratio. At different time points mitochondrial inhibitors were added as in **A** and **B**. One representative sample out of four is shown.

Table S1: CD44$^+$CD117$^+$ cells from EOC effusions show a higher tumorigenic potential.

FACS-sorted CD44$^+$CD117$^+$ cells from EOC effusions show a higher tumorigenic potential, compared to the CD44$^+$CD117$^-$ subset, when injected into RAG-2y$^{-/-}$ mice.

	**Cell number injected**		
**Cell type**	**5x10^3**	**5x10^4**	**5x10^5**
CD44$^+$CD117$^+$	7/12	-	-
CD44$^+$CD117$^-$	0/10	0/1	5/5

Table S2 : The absolute number of $CD44^{+}CD117^{+}$ cells is not affected by glucose starvation
The number of CD44/CD117 co-expressing cells in EOC effusions cultured for 14 days in the presence (+Glu) or absence (-Glu) of glucose was calculated from the percentage of $CD44^{+}CD117^{+}$ cells obtained by flow cytometry analysis of $2x10^{5}$ cells/culture condition.

	**$CD44^{+}CD117^{+}$ cell number in culture**		
**<u>Sample no.</u>**	**day0**	**day14 (+ GLU)**	**day14 (– GLU)**
**PDOVCA #11**	1950	2192	2346
**PDOVCA #12**	2260	2199	2002
**PDOVCA #13**	2000	2400	1472
**PDOVCA #14**	2000	2788	2736
**PDOVCA #15**	2400	2520	2560
**PDOVCA #17**	2200	1740	1980
**PDOVCA #27**	2346	2223	2488
**PDOVCA #29**	1093	2270	1840
**PDOVCA #32**	1375	1960	1364
**PDOVCA #39**	2900	1219	1938
**mean**	2052.4±514.8	2151.1±434.7	2072.6±456.2

P=0.88 ANOVA test

SUPPLEMENTAL METHODS

Cell lines

IGROV-1 cells were purchased from ATCC (Manassas, VA) and OC316 cells were provided by S.Ferrini (IST, Genoa, Italy); the cell lines were used within six months from resuscitation. The cells were maintained in RPMI-1640 medium (GIBCO, Invitrogen, Monza, Italy) supplemented with 10% FBS (GIBCO, Invitrogen), 1% sodium pyruvate (Lonza, Basel, Switzerland), 1% penicillin-streptomycin (Lonza) and 1 % L-glutamine (GIBCO, Invitrogen). The cells were cultured at 37°C in a humidified atmosphere containing 5% CO_2 and harvested, when 80-90% confluent, using Trypsin-EDTA (Invitrogen).

Spheroid formation assay and *in vitro* cell differentiation

To promote *in vitro* spheroid formation, EOC ascitic effusion cells were seeded in poly-2-hydroxyethyl methacrylate (PhEMA)-coated plates (BD Bioscience, Franklin Lakes, NJ) in serum-free RPMI medium supplemented with bFGF (10 ng/ml) and EGF (20 ng/ml: both from Peprotech, Rocky Hill, NJ) at a density of 2 x 10^4 cells/well. Medium was replaced every 7 days.

To determine the frequency of spheroid-forming precursors, we performed an extreme limiting dilution analysis (ELDA) in unsorted EOC cells, and FACS-sorted $CD44^+CD117^+$ and $CD44^+CD117^-$ cells. Briefly, the cells were plated at different concentrations in 96-well flat-bottom ultra-low attachment PhEMA-coated plates (BD Bioscience) in a total volume of 0.1 ml of serum-free medium RPMI medium supplemented with EGF and bFGF. Thirty replicate wells were set up for each cell concentration. After 7 days of incubation, the wells were scored for spheroid formation; the frequency of spheroid-forming precursors in each population was calculated by ELDA web tool (http://bioinf.wehi.edu.au/software/elda). Data are expressed as the number of spheroid-forming cells/10^3 cells.

The ability to differentiate *in vitro* was evaluated by plating unfractionated EOC cells, previously cultured in serum-free conditions, in the presence of 10% FBS at a density of 2 x 10^4 cells/ml. The cells were maintained at 37°C in a 5% CO_2 humidified atmosphere, and the medium replaced every 7 days.

RNA extraction, reverse transcription and gene card analysis

Total RNA was extracted by the TRIzol method according to manufacturer's instructions. cDNA was synthesized from 0.5-1 µg of total RNA with Superscript II reverse transcriptase (Invitrogen), and hybridized to TaqMan Custom Arrays specific for 62 genes (Applied Biosystems, Foster City, CA), with each sample run in triplicate. The PCR step was performed using an ABI PRISM® 7900HT Sequence Detection System (Applied Biosystems). Results were analyzed using the comparative ΔΔCt method; ΔΔCt values were utilized to calculate the $RQ=2^{-\Delta\Delta Ct}$. Data were expressed as the fold difference in gene expression (normalized to the housekeeping gene β_2-microglobulin) relative to a reference sample, as indicated in the individual figure legends. qRT-PCR efficiency ranged from 95% to 105%.

Chemotherapy sensitivity assays

To assess chemotherapy resistance, unfractionated EOC effusion cells were seeded at 2 x 10^5 cells/well in 6-well plates; in complete RPMI medium in the presence or in the absence of Doxorubicin (1 µM Ebewe Pharma, Wien, Austria); cytofluorimetric analysis was performed 48 h later.

In vivo xenograft propagation

Xenografts were generated by injecting intra-peritoneally (i.p.) 1 × 10^6 tumor cells, obtained from

primary EOC effusion samples, into severe combined immunodeficient (SCID) mice as reported elsewhere [1] Mice were purchased from Charles River (Wilmington, MA); procedures involving animals and their care were performed according to institutional guidelines that comply with national and international laws and policies (EEC Council Directive 86/609, OJ L358, 12 December 1987). About 2 months later from cell injection, animals developed tumors, which contained a predominant ascitic component.

Tumorigenicity assay

To demonstrate the tumorigenicity potential of the different EOC populations, different numbers of *ex vivo* FACS-sorted $CD44^+CD117^+$ and $CD44^+CD117^-$ cells were injected i.p. into seven-to nine-week-old Rag-2 $y^{-/-}$ mice in a total volume of 300 µl. To evaluate the tumorigenic potential of glucose-starved cells, 5 x 10^3 cells, cultured for 14 days as above in the presence or the absence of glucose, were injected i.p. into Rag-2 $y^{-/-}$ mice. At tumor establishment, mice were sacrificed, tumors harvested and isolated cells analyzed by FACS.

Western blotting (WB)

FACS-sorted $CD44^+CD117^+$ and $CD44^+CD117^-$ cells were lysed and subjected to SDS-PAGE and WB. Immunoreactivity was evaluated using the following rabbit antibodies: anti-actin (1:5,000; Sigma Aldrich, St Louise, MO), anti-PDHK1 (1:1,000; Cell Signaling, Boston, MD), anti-phospho-PDH (1:1,000; Abcam, Cambridge, U.K.), anti-PDH (1:2,000; Abcam) and anti-G6PD (1:1,000; Cell Signaling). Mouse anti-IDH2 (1:10,000) and anti-MCT4 (1:1,000) were purchased from Cell Signaling; goat anti-HKII (1:1,000) from Santa Cruz (Dallas, Texas). The blots were hybridized with a 1:5,000 dilution of HRP-conjugated anti-goat, anti-mouse or anti-rabbit antibody (Amersham-Pharmacia, Little Chalfont, U.K.), as appropriate. Finally, the signal was detected by chemiluminescence with SuperSignal kit (Pierce, Rockford, IL), and lane densitometry analyzed by standard procedures.

Mitochondrial mass analysis

To evaluate the mitochondrial mass, unfractionated EOC effusion cells were labelled with anti-CD44 and anti-CD117 antibodies, and incubated with the MitoTracker Green probe (0.1 µM; Invitrogen) for 15 min at 37°C. Results were expressed as MFI.

***In vitro* and *in vivo* 2-DG treatment**

For *in vitro* experiments unfractionated EOC effusion cells were seeded in 6-well plates at 2 x 10^5 cells/well, and treated with 6 g/L of 2-deoxiglucose (2-DG; Sigma Aldrich). At different time points (24, 48, 72h), cytofluorimetric analysis was performed to evaluate CD44/CD117 co-expression and cell viability by AnnexinV/ PI staining [2].

For *in vivo* experiments, after red blood cell lysis and lymphocyte removal, 5 x 10^5 unfractionated EOC effusion cells were injected subcutaneously (s.c.) into seven-to-nine-week-old Rag-2 $\gamma^{-/-}$ mice in 200 µl of Matrigel (BD Bioscience) in both dorsolateral flanks. When tumors reached 100 mm^3 volume, mice were divided in two groups, one receiving i.p. three times/week 2 g/kg of 2-DG, the other a same volume of saline solution. Tumor growth was evaluated by caliper measurement; when tumors of the control group reached 600 mm^3 volume, the mice were sacrificed, tumors harvested by dissection, and isolated cells analyzed by FACS.

Oxygen Consumption and Extracellular acidification rate

The oxygen consumption rate (OCR) and Extracellular acidification rate (ECAR) were determined using the SeaHorse XF Extracellular Flux Analyzer (Seahorse Biosciences, San Jose, CA) [3, 4]. Briefly, 24-well plates (XF24 V7, Seahorse Biosciences) were coated with 20 µl of Matrigel (Cultrex, Trevigen, Gaithersburg, MD) to allow cell adhesion to the bottom of the plates. Unfractioned EOC effusion cells and FACS-sorted populations were seeded at 3 x 10^4 cells/well in

complete RPMI medium, and incubated for 4 h at 37°C, 5% CO_2. The XF assay was initiated by medium replacing with DME base supplemented with 10% FBS, 1% sodium pyruvate, 1% penicillin-streptomycin, 1% glucose (Sigma Aldrich) and 1% L-glutamine. The cartridge of the instrument was loaded to dispense four different metabolic inhibitors at 20 min intervals: oligomycin (1 μM), followed by FCCP (0.6 μM for primary samples, 0.3 μM for cell lines), antimycin (1 μM) and rotenone (1 μM; all from Sigma Aldrich) over 2 h. The assay was characterized by 3 sequential measurements of OCR and ECAR at time 0 and after each reagent addition thereafter.

SUPPLEMENTAL REFERENCES

1. Indraccolo S, Tisato V, Agata S, Moserle L, Ferrari S, Callegaro M, Persano L, Palma MD, Scaini MC, Esposito G, Fassina A, Nicoletto O, Plebani M, Chieco-Bianchi L, Amadori A, D'Andrea E, et al. Establishment and characterization of xenografts and cancer cell cultures derived from BRCA1 -/- epithelial ovarian cancers. Eur J Cancer. 2006; 42(10):1475-1483.
2. Roche TE, Baker JC, Yan X, Hiromasa Y, Gong X, Peng T, Dong J, Turkan A and Kasten SA. Distinct regulatory properties of pyruvate dehydrogenase kinase and phosphatase isoforms. Prog Nucleic Acid Res Mol Biol. 2001; 70:33-75.
3. Fabian C, Koetz L, Favaro E, Indraccolo S, Mueller-Klieser W and Sattler UG. Protein profiles in human ovarian cancer cell lines correspond to their metabolic activity and to metabolic profiles of respective tumor xenografts. FEBS J. 2012; 279(5):882-891.
4. Sciacovelli M, Guzzo G, Morello V, Frezza C, Zheng L, Nannini N, Calabrese F, Laudiero G, Esposito F, Landriscina M, Defilippi P, Bernardi P and Rasola A. The mitochondrial chaperone TRAP1 promotes neoplastic growth by inhibiting succinate dehydrogenase. Cell Metab. 2013; 17(6):988-999.
